# Uterine Transplantation and Fertility Preservation: A Hopeful Horizon for Cancer Survivors

**DOI:** 10.7759/cureus.50178

**Published:** 2023-12-08

**Authors:** Deepika Dewani, Pravin Karwade, Kalyani S Mahajan

**Affiliations:** 1 Obstetrics and Gynecology, Jawaharlal Nehru Medical College, Datta Meghe Institute of Higher Education and Research, Wardha, IND; 2 Obstetrics and Gynecology, Symbiosis Medical College for Women, Symbiosis International University, Pune, IND

**Keywords:** immunosuppressive therapy, resilience, cancer, fertility, uterine transplantation

## Abstract

Uterine transplantation, a groundbreaking medical intervention, stands as a beacon of hope for cancer survivors grappling with the dual challenges of a cancer diagnosis and potential fertility loss due to aggressive treatments. This review provides a comprehensive exploration of uterine transplantation as an innovative solution for fertility preservation in the context of cancer survivorship. The multifaceted discussion encompasses the impact of cancer on fertility, the imperative of fertility preservation, and the evolution of uterine transplantation as a transformative procedure. The post-transplantation care section delves into the intricacies of recovery, the delicate balance of immunosuppressive therapy, and the ongoing support required for recipients to embrace the full spectrum of reproductive possibilities and overall well-being. Ethical considerations surrounding uterine transplantation, including donor selection, risk assessment, and societal perspectives, are critically examined to navigate the ethical landscape of this evolving field. In conclusion, uterine transplantation is presented as a medical breakthrough and a symbol of interdisciplinary collaboration, resilience, and unwavering hope. The review underscores the role of collaborative efforts among medical professionals, researchers, ethicists, and psychologists in advancing this transformative field. Looking to the future, uterine transplantation signifies a paradigm shift in fertility preservation, offering a tangible path toward parenthood for cancer survivors. The procedure, grounded in science, ethics, and compassion, illuminates the way forward, inspiring a future where fertility preservation becomes an attainable reality for those whose reproductive dreams were once compromised by cancer treatments.

## Introduction and background

Cancer, a formidable adversary to health and well-being, often inflicts collateral damage on reproductive capabilities. The aggressive nature of cancer treatments, such as chemotherapy and radiation, significantly affects the reproductive organs, jeopardizing the fertility of both male and female patients. Understanding the intricate interplay between cancer and fertility is paramount to addressing the unique challenges faced by survivors [1].

For individuals facing a cancer diagnosis, the prospect of infertility adds a layer of emotional distress. Recognizing the profound impact on quality of life, fertility preservation has emerged as a crucial aspect of comprehensive cancer care. Preserving the ability to conceive post-treatment addresses patients' immediate concerns and contributes to their long-term psychological and emotional well-being [2].

In recent years, uterine transplantation (UTx) has emerged as a groundbreaking and innovative solution to the fertility challenges posed by cancer treatments. While conventional fertility preservation methods primarily focus on gamete preservation, UTx offers a unique avenue for individuals who have lost their uterus due to cancer or other medical conditions. This review delves into the landscape of UTx, exploring its evolution, applications, challenges, and the promise it holds for cancer survivors seeking to rebuild their reproductive futures [3].

This comprehensive review aims to examine the landscape of UTx in the context of fertility preservation for cancer survivors. By synthesizing current research, successful cases, and ongoing challenges, this review seeks to provide a thorough understanding of the role UTx plays in offering hope to those whose fertility is compromised by cancer treatments. By exploring surgical techniques, post-transplant care, ethical considerations, and future directions, we aim to shed light on the potential of UTx as a transformative solution in reproductive medicine.

## Review

Methodology 

This review adopts a comprehensive approach to investigate the landscape of UTx as a fertility preservation strategy for cancer survivors. The research objective encompasses a comprehensive exploration of the impact of cancer on fertility, existing fertility preservation methods, the evolutionary trajectory of UTx, and the ethical considerations inherent to the procedure. To achieve this, a thorough search was conducted across prominent databases, including PubMed, Embase (Excerpta Medica dataBASE), Scopus, and Web of Science, up to 2023. The search employed a combination of Medical Subject Headings (MeSH) terms and keywords relevant to UTx, fertility preservation, cancer survivors, and ethical considerations. Inclusion criteria encompassed English-language, peer-reviewed primary research articles directly related to the review's scope, while exclusion criteria filtered out non-English articles, non-peer-reviewed publications, and studies not aligned with the research objectives. A two-step screening process involving independent reviews of titles/abstracts and full-text articles was executed, with discrepancies resolved through discussion.

Understanding cancer and fertility

Effects of Cancer Treatments on Reproductive Organs

Cancer treatments can profoundly impact fertility by affecting reproductive organs and endocrine health. Common cancer treatments, including chemotherapy, radiation, and surgery, have the potential to harm reproductive organs and glands, leading to either temporary or permanent infertility [4-8]. The likelihood of fertility impairment is contingent on various factors, such as the type and stage of cancer, the treatment method employed, the patient's age, and the dosage and duration of treatment [6]. Specifically, certain cancers, such as breast, uterine, cervical, ovarian, and blood cancers in women, as well as pelvic area cancers and those necessitating chemotherapy in men, carry a more substantial risk of affecting fertility [7,9]. Surgical procedures targeting cancers in the reproductive system or pelvic region can also detrimentally impact nearby reproductive tissues, influencing fertility outcomes [6].

Fertility preservation is an option for individuals facing a cancer diagnosis, and emerging research is providing clinicians with an increasing number of reproductive and hormonal management tools [8]. Patients need to discuss the potential impact of cancer treatment on fertility with their healthcare team before starting treatment and to consider options like sperm banking or egg freezing to preserve fertility [10,11]. Physicians play a crucial role in providing information about fertility preservation and reproductive options for cancer patients and survivors, and they need to understand the effects of specific cancer treatments on fertility [11].

Challenges Faced by Cancer Survivors in Preserving Fertility

Cancer survivors encounter a myriad of challenges when it comes to preserving their fertility. Time sensitivity poses a significant hurdle, as the urgency of cancer treatment often leaves little opportunity for comprehensive fertility planning. Financial constraints may limit access to fertility preservation methods, and the emotional toll of a cancer diagnosis can complicate decision-making regarding reproductive options. Moreover, the unpredictability of treatment outcomes and the potential for infertility-related distress contribute to the complexity of navigating fertility preservation for cancer survivors [10-11].

Existing Fertility Preservation Methods and Their Limitations

Existing fertility preservation methods include various options that can be applied based on a patient's age, ovarian involvement risk, available time, and cancer type. Some of the most reliable methods for premature and post-mature puberty are embryo cryopreservation and ovarian tissue cryopreservation, respectively. Other approaches, such as artificial ovary and isolation and cryopreservation of follicles, are still under investigation. These strategies combine recent advances in assisted reproductive technologies (ARTs), cryotechnologies, and novel cell culture systems [12].

Nonetheless, limitations persist, and certain methods remain in the experimental stage. For instance, ovarian transposition is classified as an experimental approach to fertility preservation [13]. The utilization of fertility preservation methods in cancer patients remains relatively underexplored, hindering a more comprehensive understanding of their efficacy [14]. Moreover, changes in legislation may impact the accessibility of specific fertility preservation options, with ovarian tissue cryopreservation currently standing as the sole available option for prepubertal patients [15]. Although fertility preservation methods provide a range of patient options, constraints exist, prompting ongoing research efforts to enhance these techniques and broaden their accessibility.

UTx: a novel approach

Definition and Explanation of UTx

UTx is a surgical procedure that involves the transplantation of a uterus from a donor to a recipient, typically a woman who has either congenital absence of the uterus (Mayer-Rokitansky-Küster-Hauser syndrome) or has lost her uterus due to a medical condition such as cancer or hysterectomy. This intricate procedure aims to restore the recipient's reproductive anatomy, enabling her to conceive and carry a pregnancy to term [16].

Historical Background and Development of UTx

The history and development of UTx can be traced back to the early 20th century. Studies in rats, domestic species, and non-human primates validated and optimized the UTx procedure in terms of surgical aspects, immunosuppression, rejection diagnosis, and peculiarities of pregnancy in immunosuppressed patients [17]. The first reported uterine transplant was performed in Saudi Arabia in 2000 when a 26-year-old woman who had suffered postpartum hemorrhage after a Cesarean section underwent the procedure [18]. The first live birth after UTx was reported in 2014 by a medical group in Sweden, involving a 35-year-old woman with a müllerian anomaly who gave birth to a premature baby [18]. In the United States, the first UTx was performed in 2016 at the Cleveland Clinic, but the patient experienced complications and required the removal of the transplanted uterus [18]. The first successful UTx in the United Kingdom took place in 2023 at the Churchill Hospital, Oxford, where doctors transplanted a uterus from a 40-year-old woman to her 34-year-old sister, who had a rare condition affecting her ability to carry a child [18]. Over the years, UTx has evolved as a potential treatment for absolute uterine factor infertility (AUFI), offering hope for women who are unable to conceive due to the absence or non-functionality of the uterus [18]. However, the procedure is still in development, and more research is needed to improve success rates and reduce risks [17].

Key Medical and Ethical Considerations in UTx

UTx raises several key medical and ethical considerations. From a medical perspective, the risks for the recipient include surgery, immunosuppression, pregnancy, and delivery [19]. Donors, recipients, partners, and unborn children must be considered in analyzing the ethical risks and benefits of UTx [19]. Additionally, the choice between brain-dead donors and living donors, as well as the risks for living donors, are important medical and ethical considerations [19,20]. Furthermore, the ethical and social value of gestation, the extension of UTx to transgender women or men, and the risks associated with immunosuppressive therapy and transplant surgery are also significant ethical considerations [21].

Ethical principles guiding other organ transplants may not directly apply to UTx, and there is general agreement on the importance of risk reduction and the sharing and maintenance of patient data on an international registry [21]. It is also acknowledged that UTx should not be offered as part of routine clinical practice until safety and efficacy are proven and more data are required to fully understand the risks and benefits of the treatment [21]. As UTx moves from the research phase to the clinical phase, it is crucial to review the ethical, legal, and social implications before widespread clinical practice [22]. The UTx presents a range of medical and ethical considerations, including the risks for recipients and donors, the ethical and social value of gestation, and the need for comprehensive risk assessment and data collection before the procedure can be widely adopted [19-22].

Candidates for UTx

Eligibility Criteria for Cancer Survivors Seeking UTx

UTx is a potential treatment for AUFI, including cases related to gynecological malignancies, offering hope for women who are unable to conceive due to the absence or non-functionality of the uterus [3,23]. The eligibility criteria for UTx include being of childbearing age (20-40 years old), having no history of HIV or hepatitis B, and having no history of major uterine surgery [23]. Additionally, potential donors must be pre-menopausal, have completed childbearing, have experienced successful pregnancy and live birth, and be genetically related to the transplant recipient [23]. However, it's important to note that UTx is still an evolving field, and more research is needed to improve success rates and reduce risks [3].

For cancer survivors seeking UTx, eligibility criteria may vary depending on the type and stage of cancer, the treatment method, and the patient's age and overall health [3]. Patients need to discuss the potential impact of cancer treatment on fertility with their healthcare team before starting treatment and to consider options like sperm banking or egg freezing to preserve fertility [3]. Physicians play a crucial role in providing information about fertility preservation and reproductive options for cancer patients and survivors, and they need to understand the effects of specific cancer treatments on fertility [3].

Psychological and Emotional Considerations for Potential Recipients

Psychological and emotional considerations weigh significantly on individuals considering UTx, as this medical procedure has profound implications for mental health, relationships, and overall well-being [24,25]. Several critical psychological and emotional aspects merit attention. The process of undergoing UTx, encompassing the surgical procedure, immunosuppression therapy, and potential risks, can induce elevated levels of stress and anxiety in recipients [25]. The inherent complexities of the transplant journey, both physical and emotional, contribute to a heightened psychological burden.

The prospect of undergoing a transplant raises concerns about body image and self-esteem among recipients, who may grapple with feelings of vulnerability and exposure due to the surgical nature of the procedure [25]. These emotional considerations underscore the need for sensitive and comprehensive support structures. The psychological impact of the transplant process is compounded by the fear of rejection, introducing feelings of failure and loss that can significantly affect the mental well-being of recipients [25]. Managing these emotional challenges becomes integral to fostering resilience and coping strategies.

UTx can strain relationships with partners, family members, and friends as the recipient and their loved ones navigate new roles and responsibilities associated with the transplant [24,25]. The impact on relationship dynamics necessitates a holistic approach to support that extends beyond the individual recipient. Recipients who successfully undergo UTx may encounter challenges in parenting and family planning, confronting fertility-related issues and potential risks associated with the transplant [24,25]. The emotional complexities surrounding these aspects require careful consideration and tailored support.

The possibility of unanticipated outcomes, such as transplant failure or complications, adds a layer to the psychological and emotional burden carried by recipients [25]. Addressing these uncertainties becomes essential in preparing individuals for the potential outcomes of the procedure. The psychological and emotional impact of UTx can persist for years as recipients and their partners grapple with ongoing challenges related to health, relationships, and fertility [24]. This underscores the need for sustained and comprehensive psychological support throughout the entire UTx journey, acknowledging the long-term nature of the emotional considerations associated with the procedure [24,25].

Screening and Selection Process for UTx Candidates

The screening and selection process for UTx candidates represents a meticulous and comprehensive evaluation designed to optimize the chances of success while minimizing potential risks [26]. This process unfolds stepwise, incorporating a series of assessments and reviews for both recipients and living donors.

Initially, candidates undergo health screening, which involves a rigorous examination through medical and psychosocial assessments. This phase may encompass the completion of questionnaires and consultations to gauge the physical and mental well-being of the individuals [26,27]. Following this, an initial selection committee reviews the candidates based on predefined criteria to determine their eligibility for further consideration [26].

The evaluation process may include in-vitro fertilization (IVF) for recipients who successfully pass the initial selection. This step aims to assess the reproductive capacity of the recipients and is an integral part of the comprehensive evaluation [26]. Subsequently, candidates undergoing the initial evaluation and IVF may proceed to a final selection committee review. This committee, composed of experts in the field, assesses the candidates further to determine their suitability for UTx [26].

The criteria for recipients and living donors typically involve blood-type compatibility, freedom from critical infectious diseases, a history of term birth, and a uterus free from significant pathologies [26]. Recipient eligibility, historically limited to otherwise healthy women with AUFI of childbearing age and deemed suitable for IVF, may evolve as UTx procedures become more widely available [26].

Crucially, healthcare professionals engaged in the UTx process play a pivotal role in providing comprehensive psychological support and counseling to potential recipients and their partners. Navigating the emotional challenges associated with UTx requires a sensitive and supportive approach, acknowledging the profound impact of the procedure on the mental well-being of those involved [28,26]. By integrating psychological support into the screening and selection process, healthcare teams contribute to a holistic care approach that addresses both the physical and emotional dimensions of UTx, fostering a supportive environment for those embarking on this transformative journey.

The surgical procedure

*Step-by-Step Explanation of the UTx* *Surgery*

The surgical procedure for UTx is a meticulously orchestrated series of steps, each crucial to the overall success of the transplantation. The process involves distinct stages, including the donor hysterectomy, the recipient operation, and the transplantation of the uterus [29,30].

The donor hysterectomy marks the initiation of the procedure, presenting two possible approaches: a lower midline laparotomy or a minimally invasive technique. In either case, the uterus, accompanied by its vascular supply, is meticulously dissected. This involves carefully identifying and preserving the bilateral uterine arteries and the inferior and superior uterine veins [30]. For cases opting for a minimally invasive approach, the graft extraction can be accomplished through either a laparotomy incision or a transvaginal route. Notably, the extracted graft undergoes immediate flushing through the bilateral uterine arteries on the back, ensuring optimal conditions for transplantation [30].

Following the donor hysterectomy, the recipient operation takes center stage, conducted through a lower midline laparotomy [30]. The pivotal aspect of this phase involves the meticulous anastomosis of the bilateral donor uterine arteries and at least one venous outflow to the recipient's internal iliac vessels. Simultaneously, the cervix of the transplanted uterus is anastomosed to the recipient's vagina, establishing the structural integration essential for the functionality of the transplanted organ [30].

The culmination of the procedure is marked by the transplantation of the uterus into the recipient's pelvis. Securing the uterus in place with sutures is a critical step, ensuring stability and integration with the recipient's anatomical structure. The ureters are stented to prevent obstruction and a pelvic drain is strategically placed [30].

Post-surgery, the recipient enters a phase of close monitoring for signs of transplant rejection and other potential complications. The introduction of immunosuppressive therapy becomes paramount at this stage, playing a pivotal role in preventing rejection of the transplanted uterus. The delicate balance between maintaining graft function and preventing rejection introduces complexities that require vigilant monitoring and potential adjustments to the immunosuppressive regimen.

Recipients may undergo IVF to enhance further the prospects of achieving pregnancy. The success of UTx is ultimately defined by a dual criterion: the sustained functionality of the transplanted organ and the successful delivery of a healthy baby [30]. This holistic approach considers both the procedure's physiological success and the recipient's broader reproductive goals, embodying the comprehensive nature of UTx as a transformative intervention in fertility preservation.

Challenges and Risks Associated With the Surgical Procedure

The surgical procedure for UTx undoubtedly holds transformative potential, yet it has its share of challenges and associated risks. These challenges encompass a spectrum of dimensions, including surgical intricacies, immunosuppressive therapy considerations, and profound psychological impacts [21,22,27,28,31].

Surgical complications: The complexity and extensiveness of the surgical procedures for both the donor and the recipient introduce a range of potential complications. Infection, thrombosis, fistula formation, ureteric injury, and genitourinary injury are recognized risks of UTx [21,27,31]. The duration of the surgery itself has raised concerns, prompting ongoing research into the utilization of robotic-assisted surgery as a potential means to reduce operative time for both donors and recipients [21].

Immunosuppressive therapy: Essential to prevent rejection of the transplanted uterus, immunosuppressive therapy introduces its own set of challenges. Recipients undergoing long-term immunosuppression may face complications such as infections and other adverse effects related to the suppression of the immune system [21,31]. Striking a delicate balance between preventing rejection and minimizing therapy-related complications remains a central focus of post-transplant care.

Psychological challenges: The psychological toll of the UTx process is a significant aspect that cannot be overlooked. Both recipients and donors may experience stress, anxiety, and concerns related to body image. The transformative nature of the procedure, coupled with the emotional nuances surrounding fertility and organ transplantation, underscores the importance of comprehensive psychological support throughout the UTx journey [21,31].

Graft complications: Graft complications pose a substantial risk, with graft loss resulting from the graft artery or vein thrombosis in the immediate postoperative period. This critical event may necessitate graft hysterectomy, adding a layer of complexity to the management of UTx outcomes [31].

Donor-related risks: Donor surgery, extending beyond a conventional hysterectomy, carries risks, particularly to the ureter. The comprehensive assessment and management of donor-related risks are crucial for ensuring the safety and well-being of individuals contributing to the UTx process [27].

Vaginal strictures: Post-surgery, recipients may face the development of vaginal strictures, a complication that may require interventions ranging from nonsurgical dilation to surgical procedures [31].

Timing of hysterectomy: Following successful transplantation, the timing of graft hysterectomy becomes a critical consideration. Various factors, including recipient/couple preference, complications of immunosuppression, and maternal/obstetrical complications, contribute to the decision-making process [31]. In addressing these challenges, thorough risk assessment and transparent communication with potential recipients and donors are imperative. Providing comprehensive support and counseling throughout the UTx process is essential to navigate these complexities and minimize their impact. Healthcare teams must remain vigilant in evaluating and addressing the multifaceted risks associated with UTx to ensure the safety, well-being, and informed decision-making of all parties involved.

Advances in Surgical Techniques and Technology

Advances in surgical techniques and technology have contributed to developing and improving UTx procedures. For example, robotic-assisted surgery has been introduced to reduce operative time for donors and recipients [21,32]. Additionally, studies in cynomolgus macaques have demonstrated that using ovarian veins reduced the operative time for living donors [33]. Other advances include minimally invasive surgery and robotics to reduce complications and the duration of surgery [21,27]. The use of uteri-ovarian veins instead of uterine veins for assuring the venous outflow in the recipient has also been explored [27]. Furthermore, advances in immunosuppressive therapy have improved the success rates of UTx procedures, reducing the risk of transplant rejection. The use of IVF has also been introduced to achieve pregnancy after UTx [32]. Overall, advances in surgical techniques and technology have contributed to the development and improvement of UTx procedures, reducing the risks and complications associated with the surgery and improving the success rates of the procedure.

Post-transplantation care

Post-Operative Recovery and Monitoring

Immediate recovery: After UTx surgery, recipients undergo a crucial phase of immediate recovery marked by vigilant monitoring and comprehensive care. This postoperative period involves a delicate balance in managing various aspects essential to the recipient's well-being. Priority is given to pain management to ensure the individual's comfort. Attention is focused on preventing infections and acknowledging the vulnerability of the recipient during this critical phase. Simultaneously, a meticulous monitoring process is implemented to assess the viability of the transplanted graft. While surgical complications are rare, their potential seriousness necessitates a proactive stance, requiring prompt intervention to preserve both the transplanted organ and the broader health of the recipient. This phase represents a pivotal time in the overall success of the procedure, demanding a multidimensional approach to recovery that extends beyond physiological aspects to encompass the intricacies of post-transplant care [29].

Psychological support: The emotional journey entwined with UTx is profound and intricate. Post-operative care recognizes the profound psychological impact, emphasizing dedicated support for recipients. Coping with the procedure involves navigating a spectrum of emotions, from the anticipation of the transplant to grappling with its success or challenges. Managing expectations becomes a vital facet of psychological support as recipients confront the evolving dynamics of their reproductive possibilities. Addressing potential stressors, whether arising from the medical complexities of the procedure or broader implications on the individual's life, is integral to a holistic approach to recovery. This psychological support extends beyond counseling; it forms a cornerstone of the comprehensive care framework. By acknowledging and addressing the emotional dimensions of the transplantation journey, healthcare professionals contribute to the immediate well-being of the recipient and the long-term psychological resilience necessary for embracing the transformative nature of UTx [34].

Immunosuppressive Therapy and its Impact on Fertility

Necessity of immunosuppression: The cornerstone of UTx success is preventing organ rejection, necessitating the crucial component of immunosuppressive therapy for recipients. These medications play a pivotal role in suppressing the recipient's immune system to mitigate the risk of rejection of the transplanted uterus. While undeniably essential for graft survival, using immunosuppressive agents introduces complexity and concern, particularly regarding their potential impact on fertility [35]. Striking a balance between the necessity of immunosuppression for organ viability and its potential consequences for reproductive capabilities becomes a central consideration in the post-transplant care regimen.

Fertility considerations: The intricate interplay between immunosuppressive agents and fertility introduces a nuanced dimension to the post-UTx landscape. Understanding the specific mechanisms by which these drugs may influence reproductive function is imperative for optimizing outcomes. The potential impact on fertility raises questions and prompts ongoing research within the field to unravel the complexities associated with immunosuppressive regimens. Exploring modifications to these regimens becomes a focus of the investigation, aiming to minimize adverse effects on fertility while maintaining the delicate balance required for successful graft integration [36]. This area of research not only contributes to the refinement of post-transplant care protocols and holds promise for enhancing the long-term reproductive possibilities for recipients.

Monitoring and adjustments: Post-transplant care involves a vigilant and dynamic process of monitoring recipients' immune function and hormonal levels. Regular assessments are imperative to gauge the individual response to the immunosuppressive regimen and to identify potential deviations from the desired balance between preventing rejection and preserving fertility. Adjustments to the immunosuppressive regimen may become necessary based on evolving medical considerations and the unique responses of individual recipients [37]. This delicate yet crucial aspect of post-transplant care demands a personalized and proactive approach, ensuring that the therapeutic balance is continually optimized to uphold both the viability of the transplanted organ and the reproductive aspirations of the recipient.

Long-Term Care and Follow-Up for UTx Recipients

Gynecological health monitoring: The commitment to long-term care after UTx involves a meticulous and vigilant approach to monitoring gynecological health. Regular examinations, including physical assessments and imaging studies, are integral to this monitoring process. These examinations aim to assess the ongoing health and functionality of the transplanted uterus, ensuring that any potential issues are identified and addressed promptly. Hormonal assessments contribute crucial data to the comprehensive evaluation, offering insights into the reproductive health of the recipient. This systematic and regular monitoring serves as a preventative measure and allows for the proactive management of any emerging gynecological concerns, reinforcing the commitment to sustained well-being [38].

Pregnancy planning and support: Long-term care extends its focus to the reproductive aspirations of UTx recipients, with a particular emphasis on pregnancy planning and support. For those aspiring to conceive, the healthcare team provides tailored support for family planning. This involves thoroughly assessing the transplanted uterus's readiness for pregnancy, considering factors such as graft health and overall reproductive function. Addressing potential obstetric challenges becomes an integral part of the long-term care plan, ensuring recipients receive comprehensive support and guidance throughout the journey to parenthood. Emotional support is woven into this aspect of care, recognizing recipients' unique challenges and aspirations in navigating the intricate path of conceiving and bringing a child into the world [39].

Quality of life considerations: Long-term care goes beyond the clinical aspects and focuses on optimizing UTx recipients' overall quality of life. This involves a multifaceted approach that addresses both physical and psychological dimensions. Lingering psychological impacts from the transplantation journey are acknowledged and addressed, ensuring that recipients receive the necessary support to navigate any emotional challenges. Supporting reproductive autonomy becomes a cornerstone, empowering recipients to make informed decisions about their reproductive futures. Additionally, integrating recipients into routine gynecological care fosters a sense of normalcy and continuity, contributing to the holistic approach to long-term well-being. By prioritizing the physiological health and the broader aspects of a recipient's life, this facet of long-term care underscores a commitment to comprehensive and patient-centered support [40].

Success stories and challenges

Notable Cases of Successful UTx

Notable cases of successful UTx have demonstrated the potential of this procedure to enable women with absolute uterine infertility to experience pregnancy and childbirth. However, challenges and limitations have also been observed. In 2014, the first successful live birth after UTx was reported in Sweden, where a 35-year-old woman with a müllerian anomaly gave birth to a premature baby [17,41]. This achievement marked a significant milestone in the field of reproductive medicine, offering hope to women facing absolute uterine factor infertility.

In the United States, the Baylor University Medical Center in Dallas, Texas, has been at the forefront of UTx research. The center reported high technical success rates, with 74% graft survival at one year and more than 80% of recipients with a viable graft at one year achieving at least one live birth [31]. This success has demonstrated the safety of UTx for the recipient, living donor, and child, providing hope for women affected by absolute uterine-factor infertility. However, it's important to note that the history of UTx includes unsuccessful cases that have highlighted the need for rigorous research to improve success rates. Until now, UTx procedures outside of specific projects have not produced healthy mothers with positive neonatal outcomes [17]. These cases underscore the potential of UTx to address absolute uterine infertility but also emphasize the need for ongoing research and careful consideration of the challenges and limitations associated with the procedure.

Challenges and Complications Faced by Recipients

Graft failure: Graft failure emerges as a significant challenge post-UTx, with a noteworthy occurrence rate of 28.8%. This complication often necessitates emergency hysterectomies due to critical factors such as thrombosis, ischemia, or infection. Graft failure represents a complex outcome, potentially influenced by various factors. It underscores the importance of meticulous postoperative monitoring to promptly identify and address issues that may compromise the viability and functionality of the transplanted uterus [42].

Surgical complications: Recipients may encounter a spectrum of surgical complications, encompassing infection, thrombosis, fistula formation, ureteric injury, and genitourinary injury. These complications, although not universal, highlight the intricacies of the surgical procedures involved in UTx. A careful balance between the technical aspects of the surgery and vigilant postoperative care is essential to mitigate the occurrence and impact of these complications, emphasizing the need for a comprehensive and individualized approach to each case [21,43].

Immunosuppressive therapy-related complications: The necessity of immunosuppressive therapy introduces a layer of complexity, with recipients requiring these medications to prevent rejection of the transplanted uterus. However, long-term immunosuppression brings challenges, including complications such as infections and other adverse effects. Balancing the imperative of preventing rejection with the potential risks associated with prolonged immunosuppression remains a central consideration in the ongoing care of UTx recipients [21,31].

Psychological challenges: The UTx process is a physical journey and a profound psychological experience for recipients. Psychological challenges, including stress, anxiety, and body image concerns, may arise as a consequence of the transplant surgery. Acknowledging and addressing these emotional dimensions are integral to the holistic care approach, emphasizing the need for psychological support and counseling throughout the transplantation journey [21,44].

Pregnancy-related complications: Despite the transformative potential of UTx, pregnancy-related complications have been reported in some cases. Issues such as placenta accreta and subchorionic hematoma underscore the complexity of achieving successful pregnancies post-transplantation. The intricate interplay between the transplanted uterus and the physiological demands of pregnancy necessitates ongoing research and careful consideration of potential complications to optimize outcomes for both the recipient and the developing fetus [31].

Delivery complications: Observations of preterm premature rupture of the fetus and other delivery-related complications further emphasize the challenges associated with UTx. These complications highlight the need for specialized obstetric care and ongoing monitoring throughout the pregnancy to navigate potential challenges and optimize delivery outcomes [31].

Ongoing Research and Improvements in UTx Outcomes

Technical success and graft survival: Research into UTx procedures has reported notably high technical success and graft survival rates. A comprehensive study involving 33 UTx recipients in the United States demonstrated a substantial 74% graft survival, providing optimistic evidence of positive outcomes in terms of technical success. These findings underscore the advancements in surgical techniques and postoperative care that contribute to the overall success of the transplantation procedure, marking a significant stride forward in the field [31].

Reproducibility and safety: Studies have consistently highlighted the reproducibility and safety of UTx, dispelling concerns that success might be confined to specific institutions. The data suggest that UTx can be implemented across multiple centers with comparable success rates, indicating that the procedure is technically feasible and safe for both the mother and child. This recognition positions UTx as a viable and increasingly accessible option within the spectrum of effective infertility treatments, fostering confidence in its potential to provide successful outcomes on a broader scale [31].

Challenges and limitations: While UTx holds promise, research has also emphasized the challenges and limitations inherent in the procedure. These include donor availability, recipient suitability, surgical complexities, and the intricate management of recipients during and after UTx, particularly in the context of subsequent pregnancies. Identifying and addressing these challenges are critical steps in refining the procedure and enhancing its overall feasibility and success [44].

Historical perspective: The history of UTx has undergone a transformative rewrite, marking the evolution of this field and the persistent dedication to rigorous research. The historical perspective reflects not only the strides made in achieving success rates that were once deemed ambitious but also the ongoing commitment to advancing UTx as a viable treatment option for individuals facing absolute uterine infertility. Acknowledging challenges and limitations becomes a driving force for continuous improvement, highlighting the need for collaborative efforts to enhance outcomes and broaden the accessibility of this transformative procedure [17].

Ethical considerations

Ethical Concerns Surrounding UTx

Donor selection and consent: Ethical considerations are central to the intricate donor selection process in UTx. Stringent examination of informed consent and voluntariness ensures potential donors fully comprehend associated risks. The ethical framework must be robust, safeguarding against coercion and upholding the donor's autonomy. Transparency, thorough communication, and a commitment to donor well-being and rights are essential [45].

Resource allocation: The scarcity of viable uterine donors raises ethical dilemmas in resource allocation. Balancing urgent recipient needs and minimizing risks to donors requires careful oversight. Ethical frameworks must guide resource allocation, prioritizing fairness, justice, and optimal resource use. Scrutiny is crucial to prevent undue burden on donors while maximizing recipient benefits [46].

Equity and access: UTx's ethical concerns extend to equitable access. Addressing socioeconomic disparities, geographic accessibility, and potential commercialization is vital. Ethical considerations involve strategies to ensure fair access regardless of financial means or location. A conscientious approach is imperative to prevent healthcare disparities, making UTx a pathway to reproductive possibilities for all [21].

Balancing the Benefits and Risks for Both Donors and Recipients

Recipient risks: Ethical considerations demand a meticulous examination of the risks borne by recipients undergoing UTx. This encompasses not only the physical implications of the surgical procedure but also the potential psychological impact and the long-term consequences of immunosuppressive therapy. Informed consent processes play a pivotal role in this ethical landscape, requiring transparent communication of these risks to empower recipients in making well-informed decisions about their reproductive futures. The ethical imperative is ensuring recipients are cognizant of the potential challenges and uncertainties associated with UTx, allowing them to navigate the decision-making process with autonomy and clarity [47].

Donor risks: The altruistic nature of uterine donors underscores the ethical responsibility to safeguard their well-being. Ethical scrutiny must be applied to balance the potential risks associated with the surgical donation process against the desire to contribute to another individual's fertility journey. This involves ongoing ethical evaluation and refinement of donor protocols to minimize risks and ensure that the donation process aligns with principles of voluntariness, autonomy, and the protection of donor welfare. Ethical considerations extend beyond the immediate act of donation, requiring a commitment to the long-term well-being of those who participate in this altruistic endeavor [48].

Benefit assessment: Ethical evaluation in UTx necessitates a nuanced consideration of the benefits conferred upon both recipients and donors. Beyond the immediate joy associated with successful transplantation and potential pregnancies, an ethical framework should extend its assessment to encompass the long-term physical, psychological, and societal benefits. This holistic evaluation is crucial for ethical decision-making, ensuring that the potential benefits are weighed against the associated risks and that the outcomes align with principles of justice, fairness, and the promotion of overall well-being [49].

Societal Perspectives on UTx for Fertility Preservation

Cultural attitudes and beliefs: The ethical landscape of UTx is intricately woven with diverse cultural attitudes and beliefs. Societal perspectives on this medical intervention vary significantly across cultures and communities, influencing the reception and acceptance of the procedure. Ethical considerations involve respecting cultural diversity, acknowledging different belief systems, and promoting inclusivity in UTx discourse. A careful balance is required to navigate the intersection of medical advancements and cultural contexts, ensuring that ethical practices are culturally sensitive and responsive to the diverse perspectives that shape societal attitudes [50].

Media influence: The portrayal of UTx in the media substantially influences public opinion. Ethical considerations include media outlets' responsibility to present accurate information, avoid sensationalism, and contribute to an informed public discourse on the ethical implications of this evolving medical intervention. Transparency, clarity, and responsible reporting are crucial to mitigate misinformation and foster a public understanding that aligns with the ethical dimensions of UTx. The media plays a pivotal role in shaping societal attitudes, and ethical practices in reporting contribute to an informed and engaged public [21].

Legal and policy frameworks: Societal acceptance of UTx often finds reflection in legal and policy frameworks. Ethical considerations in this realm involve evaluating the adequacy of existing regulations, ensuring the protection of the rights and well-being of donors and recipients, and addressing any legal gaps that may emerge as UTx becomes more commonplace. The ethical imperative lies in establishing and maintaining legal frameworks that not only safeguard the interests of those involved but also foster a societal environment that is supportive and protective of ethical practices in UTx [51].

## Conclusions

In conclusion, while acknowledging the transformative hope that UTx brings, it is paramount that we translate this optimism into concrete action. To propel this field forward and ensure wider accessibility, we urgently call for increased financial support for cutting-edge research. Adequate funding will enable the development of more effective immunosuppressive drugs, essential for refining the transplantation process and minimizing potential risks. Furthermore, there is an imperative need to establish comprehensive support systems tailored to the unique needs of UTx recipients. This includes not only medical care but also robust psychological and emotional support throughout the entire journey. By rallying behind these specific initiatives, we can actively contribute to the advancement of UTx, making this groundbreaking procedure not just a beacon of hope but a tangible reality for countless individuals. Together, let us pave the way for a future where the benefits of UTx are within reach for all those in need, fostering a landscape of enhanced medical care, research innovation, and unwavering support for those on this transformative path.
